# Supplementary material for: Age-related matrix stiffening epigenetically regulates α-Klotho expression and compromises chondrocyte integrity
Source: Nat Commun. 2023 Jan 10;14:18. doi: 10.1038/s41467-022-35359-2 (PMC9832042; doi:10.1038/s41467-022-35359-2)
Supplement: Supplementary file 1 — Supplementary Information [file 41467_2022_35359_MOESM1_ESM.pdf]

Supplementary Online Content

Age-related matrix stiffening epigenetically regulates α-Klotho expression and compromises chondrocyte integrity

†Hirotaka Iijima[1,2,3], Gabrielle Gilmer[4-8], Kai Wang[1,7,8], Allison C. Bean[1,9], Yuchen He[10], Hang Lin[5,9,10], Wan-Yee Tang[11], Daniel Lamont[12], Chia Tai[13], Akira Ito[13], Jeffrey J Jones[14], Christopher Evans[15], *Fabrisia Ambrosio[1,5,7-11]

[1]Department of Physical Medicine and Rehabilitation, University of Pittsburgh, Pittsburgh, PA
[2]Japan Society for the Promotion of Science, Tokyo, Japan
[3]Institute for Advanced Research, Nagoya University, Nagoya, Japan
[4]Medical Scientist Training Program, School of Medicine, University of Pittsburgh, Pittsburgh, PA
[5]Department of Bioengineering, University of Pittsburgh, Pittsburgh, Pittsburgh, PA
[6]Cellular and Molecular Pathology Graduate Program, University of Pittsburgh, Pittsburgh, PA
[7]Discovery Center for Musculoskeletal Recovery, Schoen Adams Research Institute at Spaulding, Boston, MA
[8]Department of Physical Medicine & Rehabilitation, Harvard Medical School, Boston, MA
[9]McGowan Institute for Regenerative Medicine, University of Pittsburgh, Pittsburgh, PA
[10]Department of Orthopaedic Surgery, University of Pittsburgh, Pittsburgh, PA
[11]Department of Environmental and Occupational Health, University of Pittsburgh School of Public Health, Pittsburgh, PA
[12]Petersen Institute of Nanoscience and Engineering, University of Pittsburgh, Pittsburgh
[13]Department of Motor Function Analysis, Human Health Sciences, Graduate School of Medicine, Kyoto University, Kyoto, Japan
[14]Proteome Exploration Laboratory, Beckman Institute, California Institute of Technology, Pasadena, CA
[15]Department of Physical Medicine & Rehabilitation, Mayo Clinic, Rochester, MN

*Corresponding author. fambrosio@mgh.harvard.edu, †Co-corresponding author. iijima@met.nagoya-u.ac.jp

The PDF file includes:

Figure S1. Computational histological analysis for the quantification of cartilage surface roughness
Figure S2. Multi-omics analysis of mass spectrometry and RNA-seq identified the PI3K/Akt signaling pathway as a driver of age-related cartilage degeneration
Figure S3. Ingenuity pathway analysis identified INS and INSR as regulators of cartilage aging
Figure S4. Inhibition of α-Klotho activates PI3K/Akt signaling
Figure S5. α-Klotho expression decreases with increased cartilage degeneration assessed by OARSI score in a sex-dependent manner
Figure S6. Genetic inhibition of α-Klotho (*Klotho*$^{+/-}$) triggered cartilage surface disruption
Figure S7. Relationship between lamins, nuclear eccentricity, and α-Klotho in murine cartilage
Figure S8. Decreased matrix stiffness drives aged chondrocytes towards a young phenotype
Figure S9. Matrix stiffness regulates lamin A/C expression
Figure S10. Stiff substrates alter chondrocytes cellular morphology
Figure S11. Sensitivity analysis for stiffness-dependent regulation on chondrogenicity
Figure S12. Epigenetic regulation of α-Klotho by matrix stiffness in aged chondrocytes
Figure S13. Chromatin immunoprecipitation (ChIP) analyses of chondrocytes cultured on polyacrylamide gels with different stiffness
Figure S14. Impact of siRNA treatment for *Dnmt1* on nuclear morphology
Figure S15. Latrunculin A treatment on aged chondrocytes
Figure S16. Inhibition of mechanotransduction in aged chondrocytes
Figure S17. Direct biological impact of BAPN administration on aged chondrocytes

Table S1. Description of top 10 nuclear morphological features significantly altered by aging
Table S2. Preparation and characterization of pAAm hydrogels
Table S3. Information and dilution protocol of antibodies

Supplemental references

This supplementary material has been provided by the authors to give readers additional information.

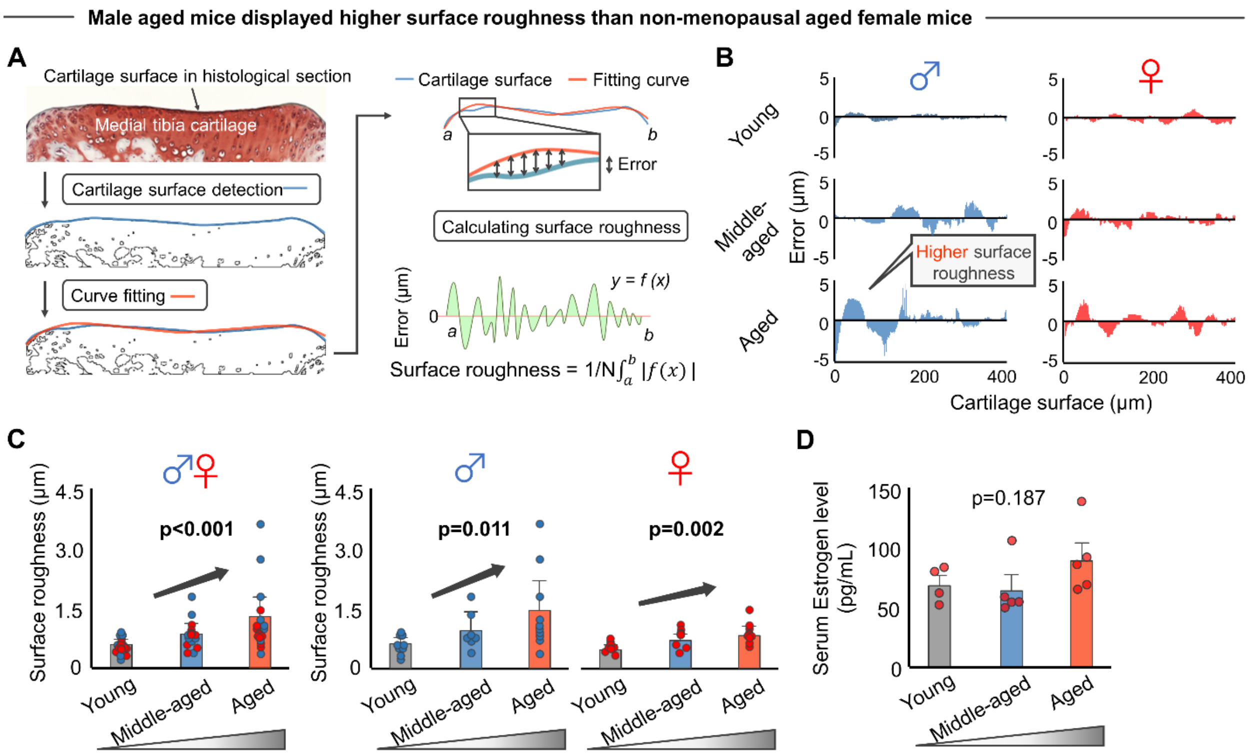

Figure S1. Computational histological analysis for the quantification of cartilage surface roughness

A. Computational analysis for calculation of cartilage surface roughness. Surface roughness was calculated as the deviation between actual cartilage surface (blue solid line) and fitted curve applied to the cartilage surface (red solid line). **B.** Representative error value between fitting curve and cartilage surface. **C.** Aging induced progressive surface roughness in murine medial tibial plateaus in a sex-dependent manner (male, n = 10 for young and aged, n = 7 for middle-aged; female, n = 10 for young and aged, n = 9 for middle-aged). **D.** Serum estrogen levels were similar across the three age groups in female mice (n = 4 for young, n = 5 for middle-aged and aged). Statistical analyses were performed using linear regression analysis (**C**) or one-way ANOVA (**D**). Data are presented as means ± 95% confidence intervals. Source data are provided as a Source Data file.

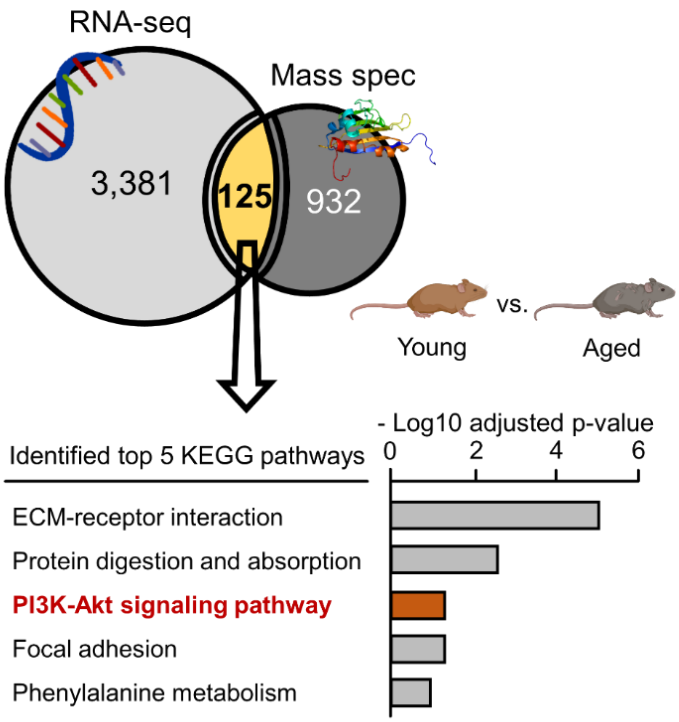

Figure S2. Multi-omics analysis of mass spectrometry and RNA-seq identified PI3K/Akt signaling pathway as a driver of age-related cartilage degeneration

We accessed archived RNA-seq data assessing global transcriptomic changes across the lifespan of mice[1]. KEGG enrichment analysis from overlapped 125 genes identified PI3K/Akt signaling pathway within the top 5 changing pathways. Portions of the figure were created with biorender.com. Source data are provided as a Source Data file.

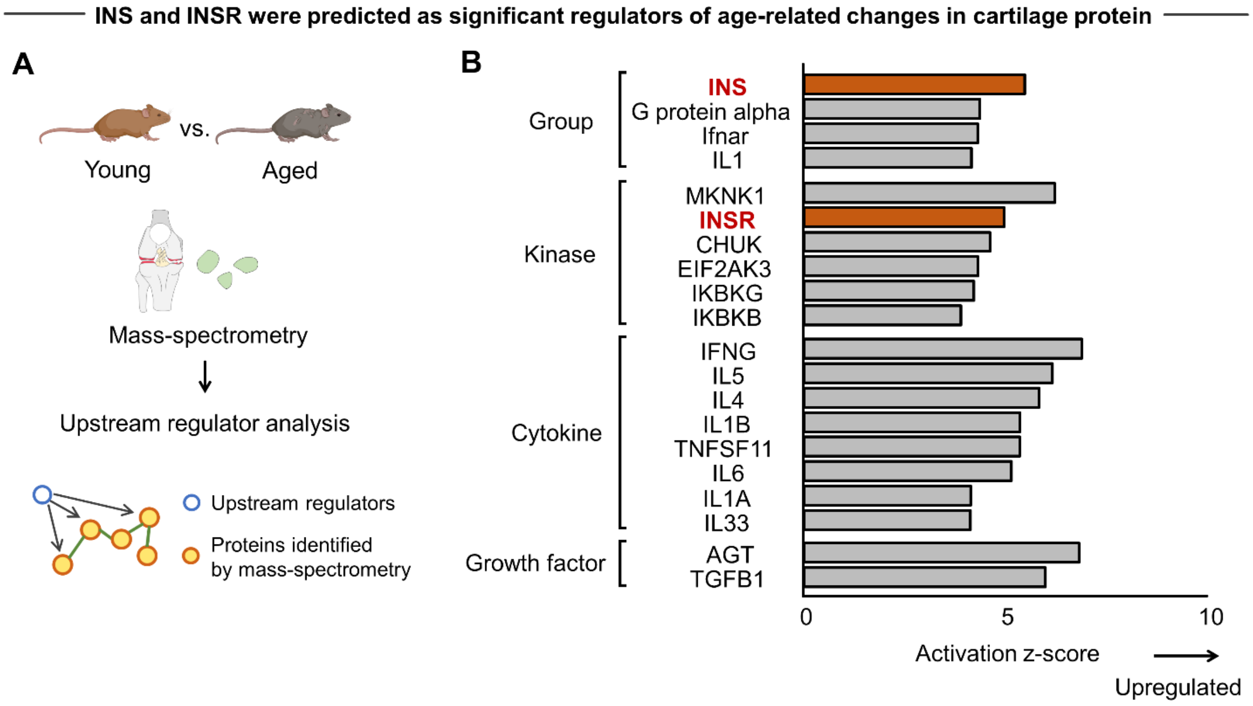

Figure S3. Ingenuity pathway analysis identified INS and INSR as regulators of cartilage aging

A. Schematic showing the analytical flow of upstream regulator analysis with mass-spectrometry proteomics data obtained by male young and aged mice. **B.** Upstream regulator analysis identified insulin (INS) and insulin receptor (INSR) as significant regulators upregulated in the cartilage of aged mice. Graph shows the top 20 upregulated upstream regulators. Portions of the figure were created with biorender.com. Source data are provided as a Source Data file.

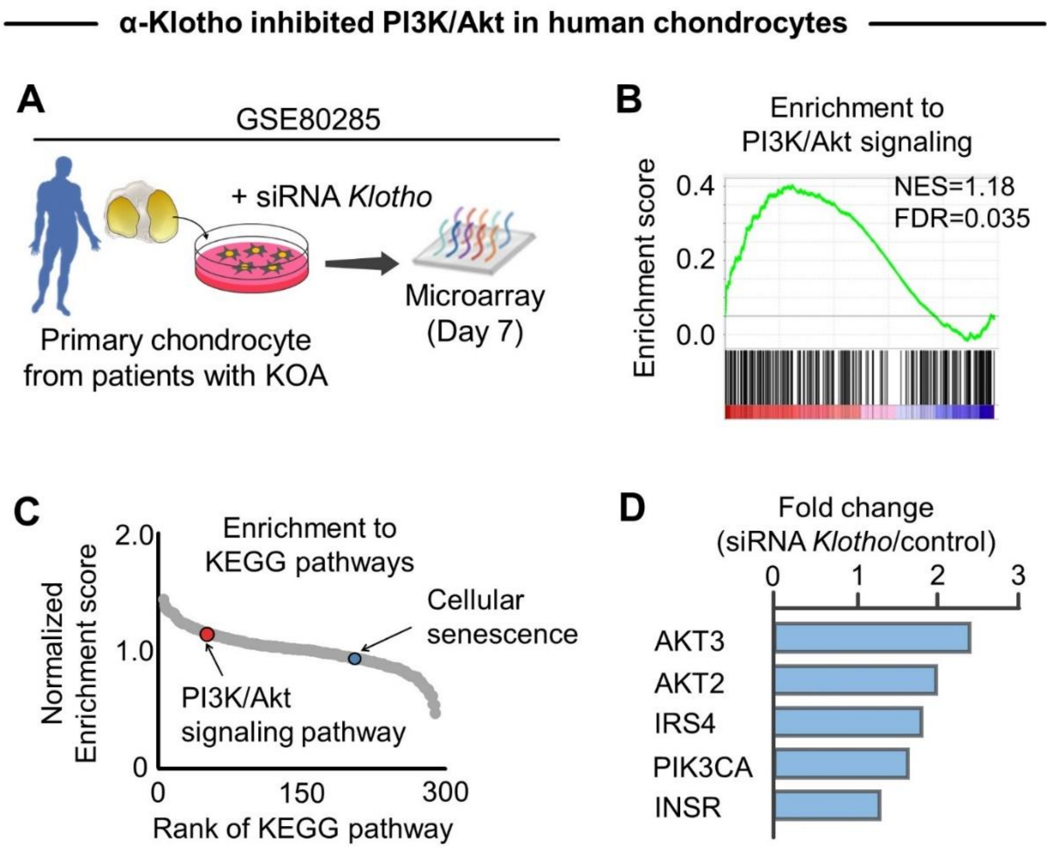

Figure S4. Inhibition of α-Klotho activates PI3K/Akt signaling

A. Schematic showing the experimental flow of microarray from human primary OA chondrocytes that is publicly available (Chuchana P[2]; GSE80285). **B.** Gene set enrichment analysis plots for PI3K/Akt gene set showing significant changes following treatment with siRNA *Klotho* (FDR = 0.035). FDR: false discovery ratio. NES: normalized enrichment score. **C.** KEGG pathways were ranked on the basis of NES; the PI3K/Akt signaling pathway and cellular senescence are highlighted in red and blue as an illustration purpose, respectively. **D.** Key elements of Insulin/PI3K/Akt pathway showing that α-Klotho inhibits Insulin/PI3K/Akt signaling in human KOA chondrocytes. Log10 fold change (FC) in expression is provided relative to control treatment. Portions of the figure were created with biorender.com. Source data are provided as a Source Data file.

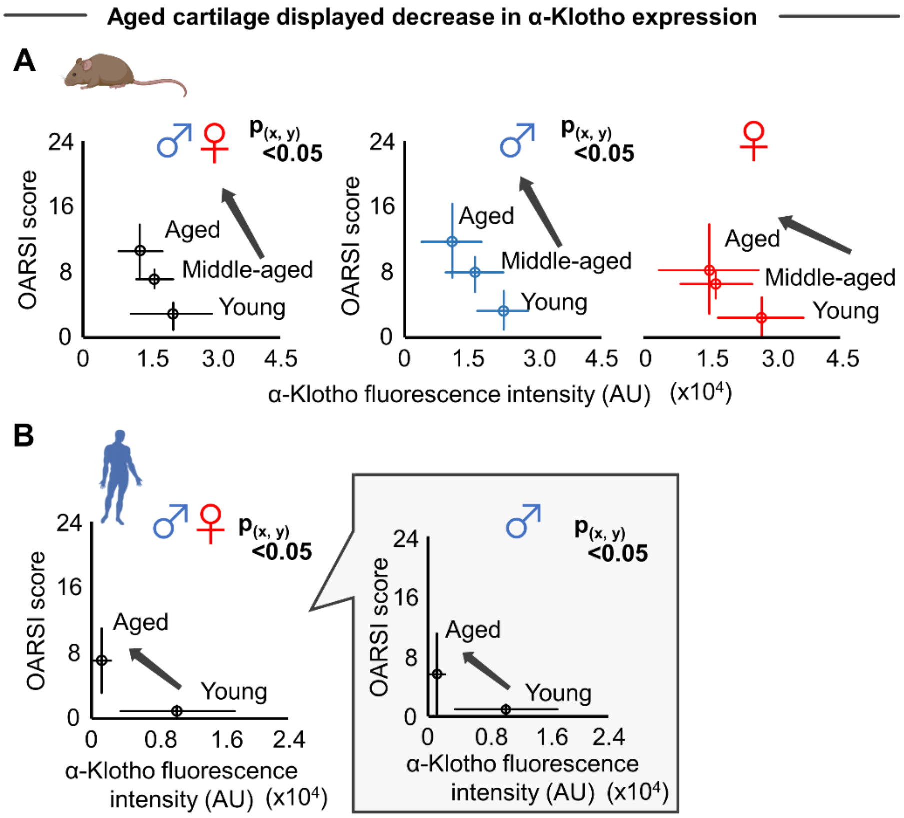

Figure S5. α-Klotho expression decreases with increased cartilage degeneration assessed by OARSI score in a sex-dependent manner

A. Murine samples (n = 5/sex/age). **B.** Human samples (n = 7/group). Statistical analyses were performed using linear regression analysis (**A**) or two-tailed Student *t*-test (**B**). P(x,y)<0.05 indicates a statistically significant relationship between aging and α-Klotho (x-axis) and aging and OARSI score (y-axis) which was confirmed by linear regression (**A**) and a two-tailed Student *t*-test (**B**). Data are presented as means ± 95% confidence intervals. Portion of the figure were created with biorender.com. Source data are provided as a Source Data file.

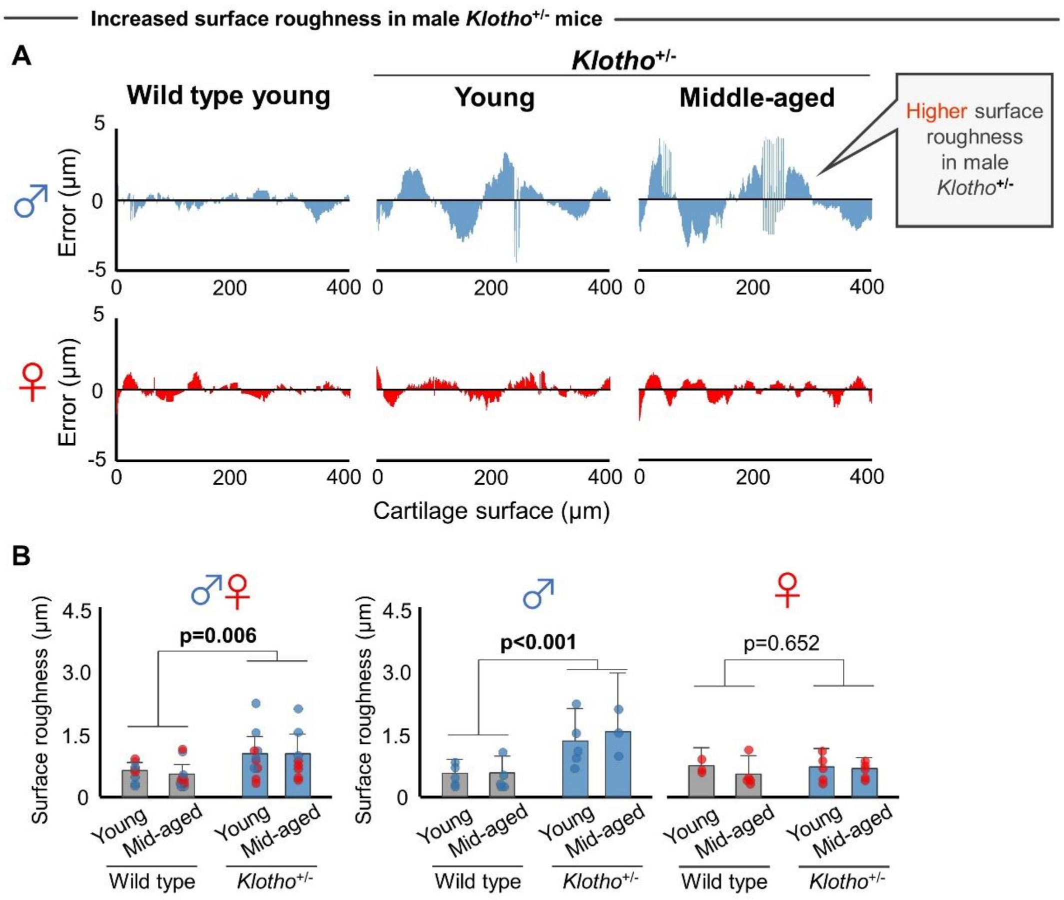

Figure S6. Genetic inhibition of α-Klotho (*Klotho*$^{+/-}$) triggered cartilage surface disruption

A. Representative error value between the fitting curve and cartilage surface in wild type and *Klotho*$^{+/-}$ mice. **B.** Cartilage in *Klotho*$^{+/-}$ male mice displayed higher surface roughness (young, n = 7 for wild type [3 females], n = 10 for *Klotho*$^{+/-}$ [5 females]; middle-aged, n = 10 for wild type [5 females], n = 8 for *Klotho*$^{+/-}$ [5 females]). Statistical analysis was performed using two-way ANOVA (**B**). Data are presented as means ± 95% confidence intervals. Source data are provided as a Source Data file.

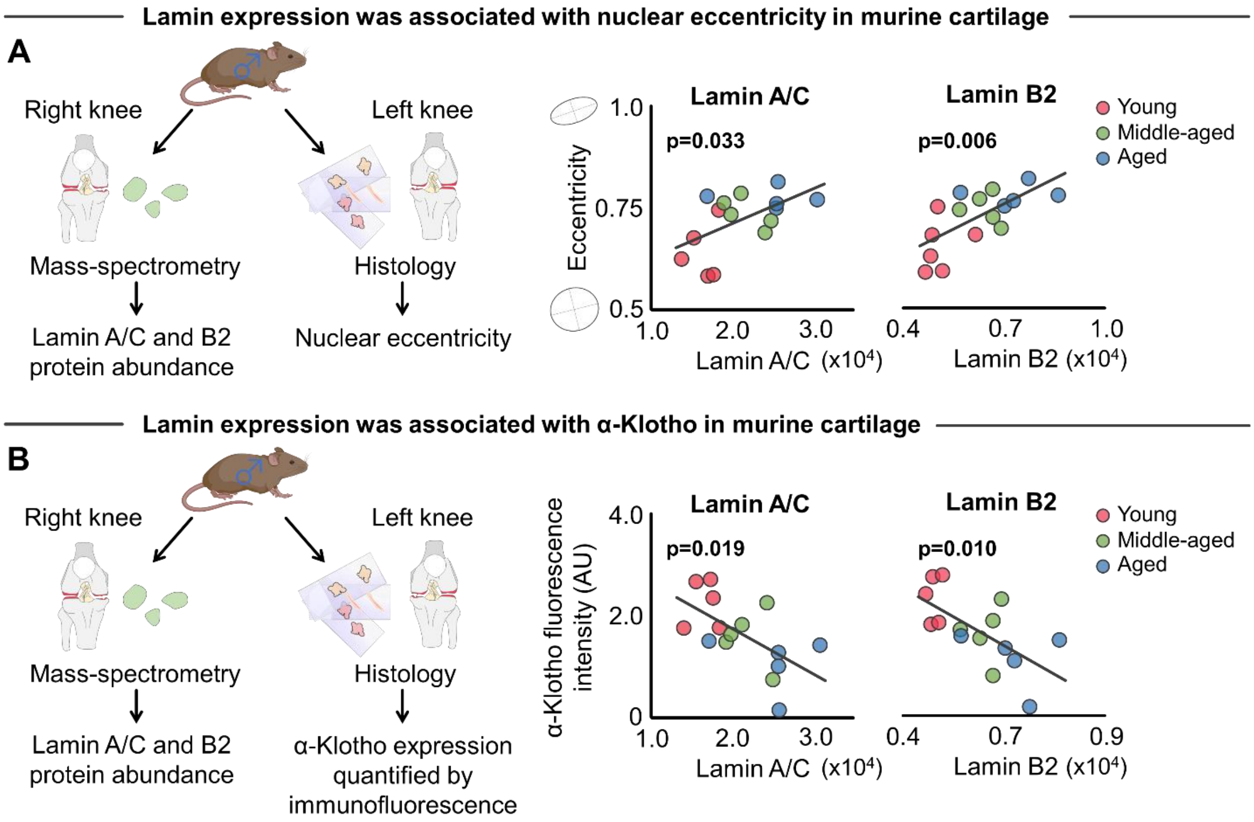

Figure S7. Relationship between lamins, nuclear eccentricity, and α-Klotho in murine cartilage

A. Lamin expression and nuclear eccentricity in murine cartilage across the lifespan was determined from mass spectrometry-based proteomics (right knee) and histological assessment (left knee), respectively. Nuclear eccentricity was significantly associated with lamin A/C and lamin B2 protein abundance. Nuclear eccentricity was quantified by the CellProfiler software using DAPI staining (n = 5/age; 30-80 nuclei per individual mouse). **B.** Lamin and α-Klotho expression in murine cartilage across the lifespan was determined from mass spectrometry-based proteomics (right knee) and histological assessment (left knee), respectively. Age-related declines in α-Klotho were significantly associated with increased lamin A/C and lamin B2 protein abundance. α-Klotho expression per cell was quantified using immunofluorescence (50-100 cells per individual mice). Statistical analyses were performed using linear regression (n = 5/age). Portions of the figure were created with biorender.com. Source data are provided as a Source Data file.

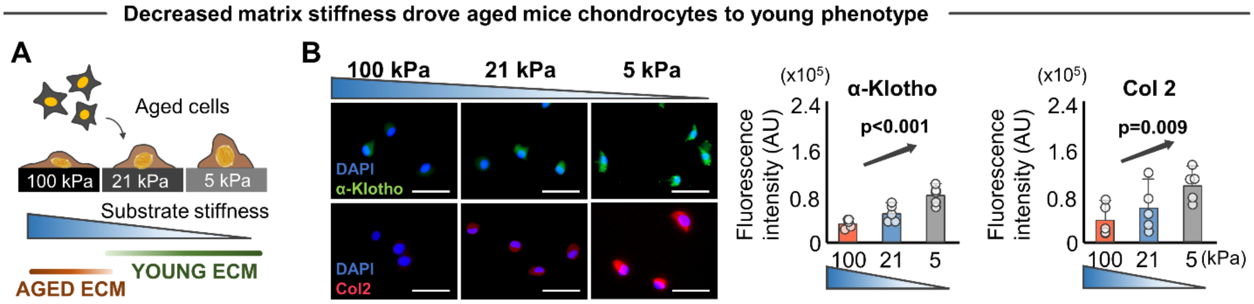

Figure S8. Decreased matrix stiffness drives aged chondrocytes towards a young phenotype

A. Schematic showing the experimental protocol. Primary chondrocytes isolated from aged knee joint cartilage were seeded onto polyacrylamide gels engineered to mimic a physiological range of knee cartilage matrix stiffnesses (5kPa, 21kPa, and 100kPa). **B.** Soft substrates increased α-Klotho and type II collagen in aged chondrocytes quantified by immunofluorescence (n = 5/group; 30-70 cells per individual sample). Statistical analyses were performed using linear mixed effect model (**B**). Data are presented as means ± 95% confidence intervals. Source data are provided as a Source Data file.

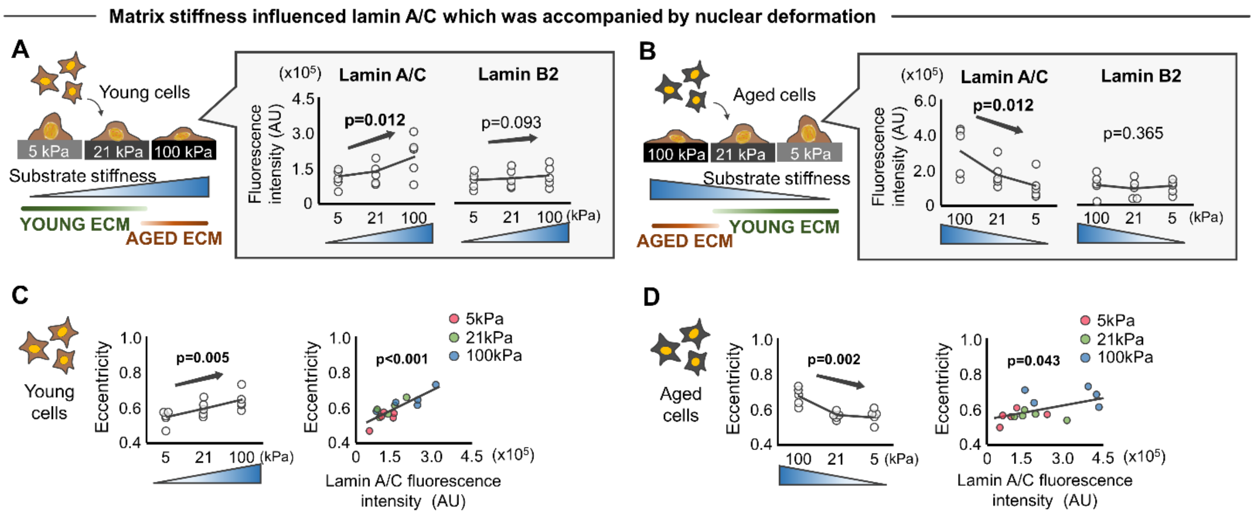

Figure S9. Matrix stiffness regulates lamin A/C expression

A. Stiff substrates increased lamin A/C, an indicator of nuclear stiffness, but not lamin B2 in young chondrocytes quantified by immunofluorescence (n = 5/group; 10-20 cells per individual sample). **B.** Soft substrates decreased lamin A/C, an indicator of nuclear stiffness, but not lamin B2 in aged chondrocytes, as quantified by immunofluorescence (n = 5/group; 10-20 cells per individual sample). **C.** Stiff substrates increased nuclear eccentricity (i.e., less roundness) in young chondrocytes, which was significantly associated with lamin A/C expression (n = 5/group). **D.** Soft substrates decreased nuclear eccentricity (i.e., more roundness) in aged chondrocytes, which was significantly associated with lamin A/C expression (n = 5/group). Statistical analyses were performed using linear mixed effect model (**A-D**) or linear regression (**C-D** for the correlation between lamin A/C and eccentricity). Data are presented as means ± 95% confidence intervals. Source data are provided as a Source Data file.

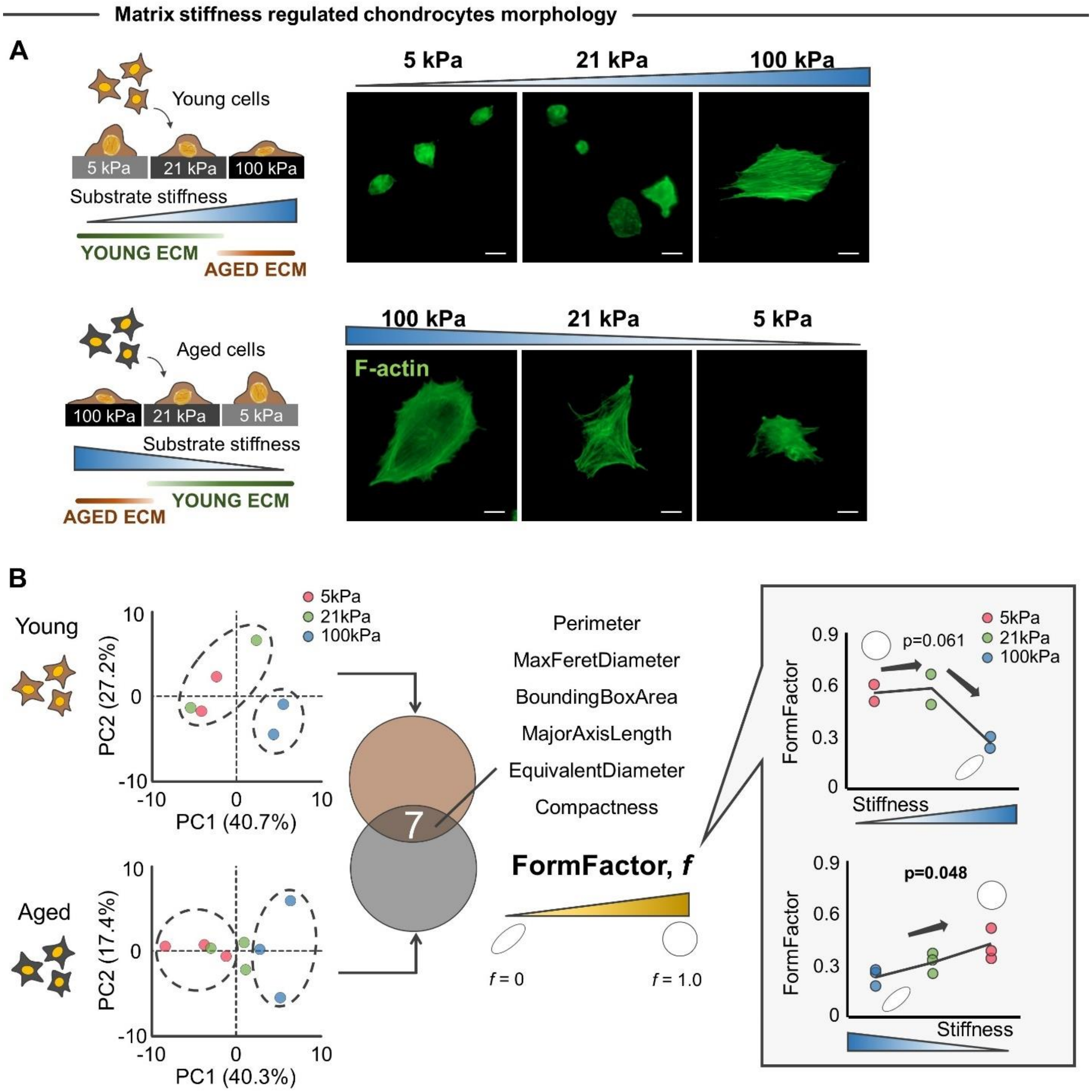

Figure S10. Stiff substrates alter chondrocytes cellular morphology

A. Primary chondrocytes from young (n = 2/group) and aged (n = 3/group) mice cultured within a physiological range of knee cartilage ECM stiffnesses (5kPa, 21kPa, and 100kPa) displayed differing morphologies. Representative F-actin image of cells on gels of each stiffness. Scale bar: 20μm. **B.** Principal component analysis (PCA) of 53 cell morphological variables showed separate clusters between soft and stiff substrates. Form factor (i.e., roundness) was one feature sensitive to the stiffness of substrates in both young and aged chondrocytes (**B**). Statistical analyses were performed using linear mixed effect model (**B**). Source data are provided as a Source Data file.

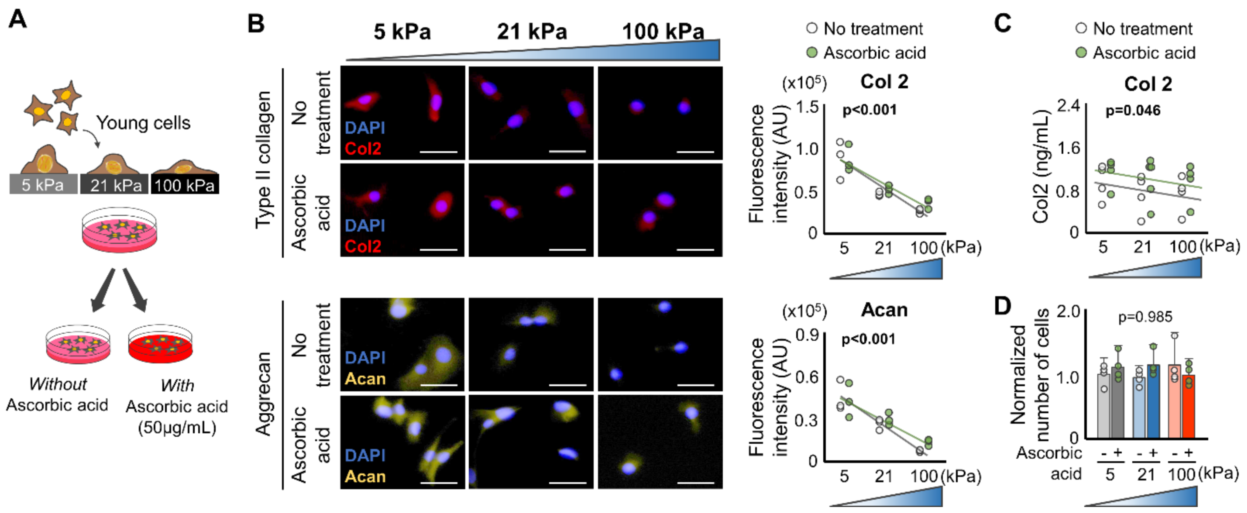

Figure S11. Sensitivity analysis for stiffness-dependent regulation on chondrogenicity

A. Schematic showing the experimental protocol. Primary chondrocytes isolated from young murine cartilage were seeded onto polyacrylamide gels engineered to mimic a physiological range of cartilage matrix stiffness (5kPa, 21kPa, and 100kPa) and cultured with and without ascorbic acid (50µg/mL). **B.** Stiff substrates reduces chondrogenicity markers (type II collagen and aggrecan) in young chondrocytes independent of the presence of ascorbic acid. Data was quantified by immunofluorescence (n = 5/group; 30-70 cells per individual sample). Scale bar: 50µm. **C.** Enzyme-linked immunoassay (ELISA) for soluble type II collagen in conditioned medium. Chondrocytes cultured on stiff substrates released significantly less soluble type II collagen compared to chondrocytes cultured on soft substrates independent of the presence of ascorbic acid. **D.** The number of chondrocytes did not change significantly with substrate stiffness (5, 21, and 100kPa) or the presence of ascorbic acid. Statistical analyses were performed using linear mixed effect model (**B**, **C**) and analysis of variance (**D**). Since there was no significant interaction effect based on the presence of ascorbic acid (**B**; p = 0.509 for type II collagen, p = 0.280 for aggrecan) and ELISA (**C**; p = 0.620), data of no treatment and ascorbic acid groups was combined for the statistical analysis with linear mixed effect model. Data are presented as means ± 95% confidence intervals. Portions of the figure were created with biorender.com. Source data are provided as a Source Data file.

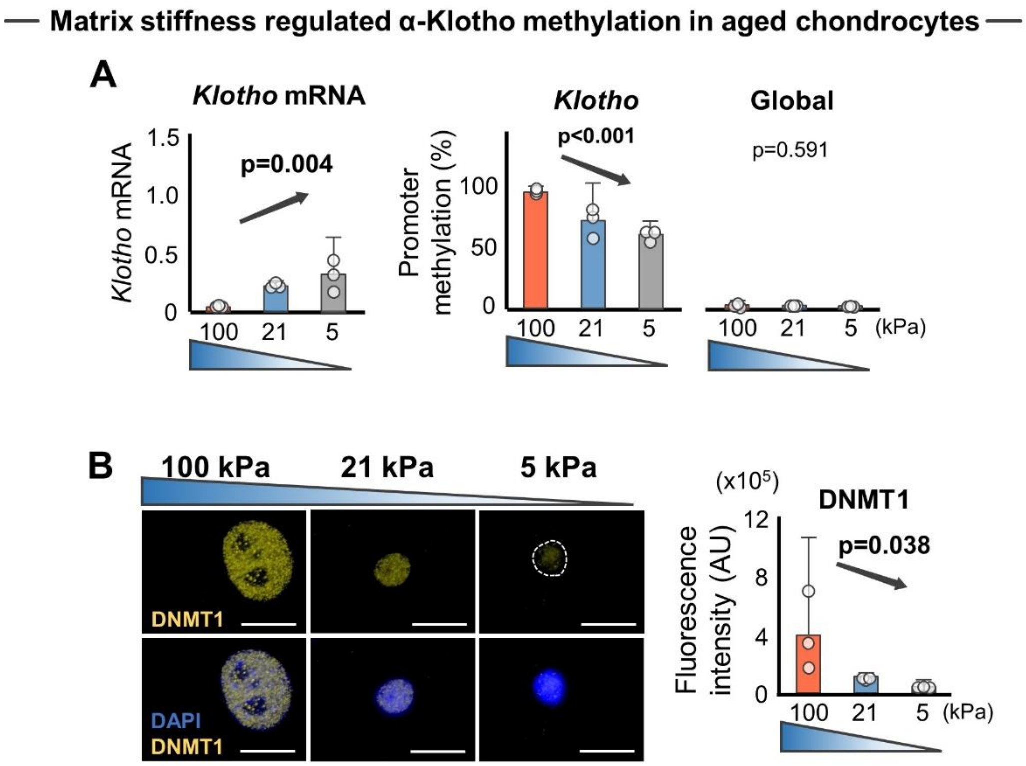

Figure S12. Epigenetic regulation of α-Klotho by matrix stiffness in aged chondrocytes

A. Soft substrates upregulated α-Klotho gene expression and decreased *Klotho* promoter methylation as well as global DNA methylation in aged chondrocytes (n = 3/group). **B.** Soft substrates decreased DNMT1 expression in aged chondrocytes (n = 3/group). Scale bar: 20μm. Statistical analyses were performed using linear mixed effect model (**A**, **B**). Data are presented as means ± 95% confidence intervals. Source data are provided as a Source Data file.

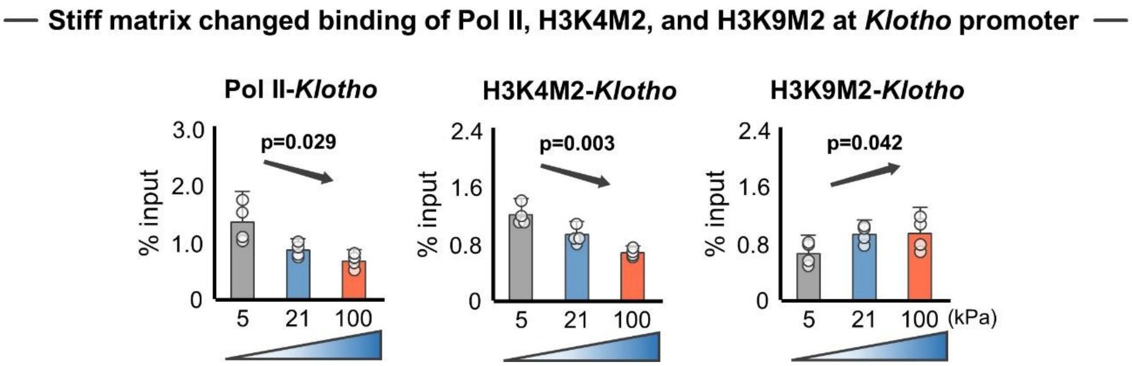

Figure S13. Chromatin immunoprecipitation (ChIP) analyses of chondrocytes cultured on polyacrylamide gels with different stiffness

Stiff substrates decreased binding of RNA Polymerase II (Pol II), active chromatin mark H3K4M2, but increased binding of a repressive chromatin mark H3K9M2 at *Klotho* promoter in young chondrocytes quantified by ChIP analyses (n = 4/group). Statistical analyses were performed using linear mixed effect model. Data are presented as means ± 95% confidence intervals. Source data are provided as a Source Data file.

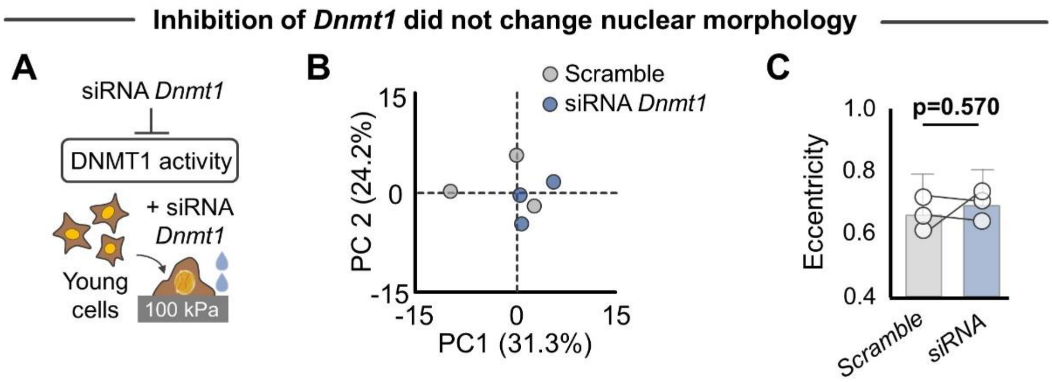

Figure S14. Impact of siRNA treatment for *Dnmt1* on nuclear morphology

A. Schematic showing the experimental protocol. Nuclear morphology of young chondrocytes was quantified using CellProfiler after treatment of siRNA for *Dnmt1*. **B.** Principal component analysis showing a similar nuclear morphological profile in chondrocytes treated by non-targeting scramble (i.e., control) and siRNA for *Dnmt1* (n = 3/group; 10-20 nuclei per sample). **C.** siRNA for *Dnmt1* did not significantly change nuclear eccentricity quantified by the CellProfiler software using DAPI staining (n = 5/age; 15-30 nuclei per individual mouse). Statistical analyses were performed using two-tailed paired *t*-test (**C**). Data are presented as means ± 95% confidence intervals. Source data are provided as a Source Data file.

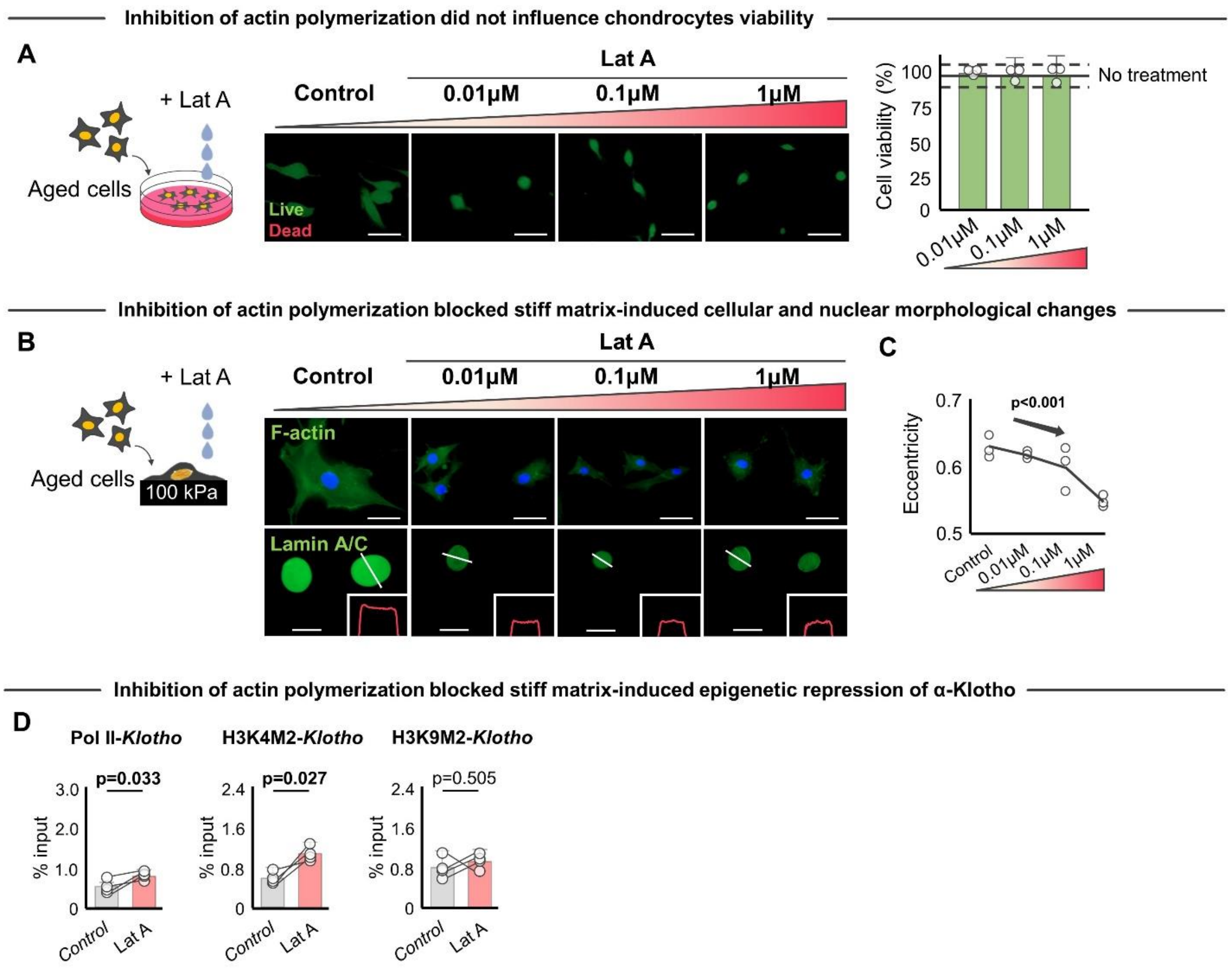

Figure S15. Latrunculin A treatment on aged chondrocytes

A. Different concentrations (0.01µM, 0.1µM, 1.0µM) of Latrunculin A (Lat A) did not influence the viability of aged chondrocyte (n = 3/group). Scale bar: 50µm. **B.** Lat A treatment of aged chondrocytes cultured on stiff substrates altered stress fiber formation and lamin A/C expression. Insets represent fluorescence intensity of lamin A/C at nuclear midsection level (white line). Scale bar: 50µm (F-actin) and 20µm (lamin A/C). **C.** Lat A treatment altered nuclear eccentricity in a dose-dependent manner. Nuclear eccentricity was quantified by CellProfiler software using DAPI staining (n = 3/group; 20-30 nuclei per sample). **D.** Inhibition of actin polymerization blocked decreases in binding of RNA Polymerase II (Pol II), and active chromatin mark H3K4M2, but not the repressive chromatin mark, H3K9M2, at the *Klotho* promoter in aged chondrocytes quantified by chromatin immunoprecipitation (ChIP) analyses (n = 4/group). Statistical analyses were performed using an analysis of variance (**A**), linear mixed effect model (**C**), and two-tailed paired *t*-test (**D**). Portions of the figure were created with biorender.com. Source data are provided as a Source Data file.

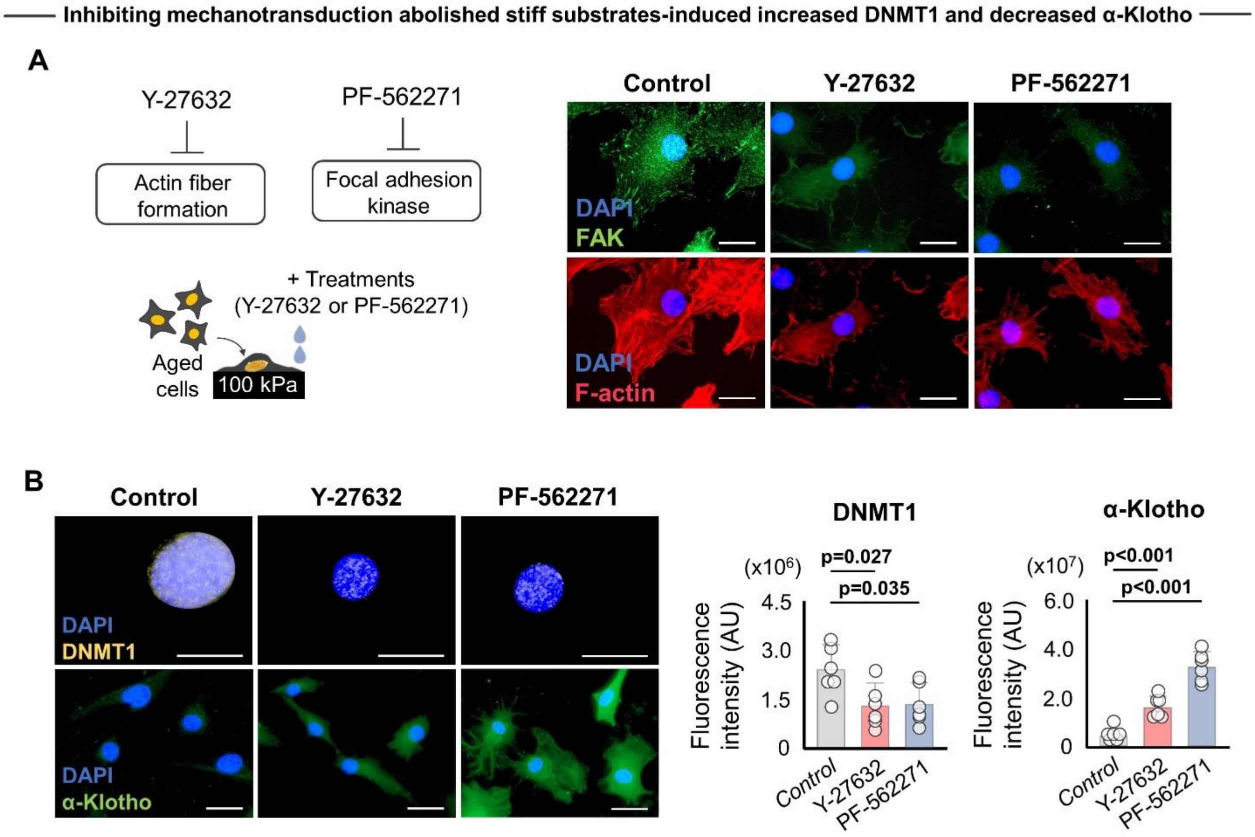

Figure S16. Inhibition of mechanotransduction in aged chondrocytes

A. Schematic showing the experimental protocol. Aged murine chondrocytes cultured on stiff substrate were treated with Y-27632, inhibitor of actin fiber formation, as well as PF-562271, an inhibitor of focal adhesion kinase (FAK). Immunofluorescence images for FAK and F-actin were shown as a validation purpose, which was reproducible in another set of images. FAK expression was decreased after Y-27632 and PF-562271 treatments and chondrocytes treated by both drugs displayed altered stress fiber formation. Scale bar: 20μm. **B.** Both treatments abolished stiff substrates-induced increased DNMT1 level and decreased α-Klotho level (n = 6/group). Scale bar: 20μm. Statistical analyses were performed using a Dunnett's test (**B**). Data are presented as means ± 95% confidence intervals. Source data are provided as a Source Data file.

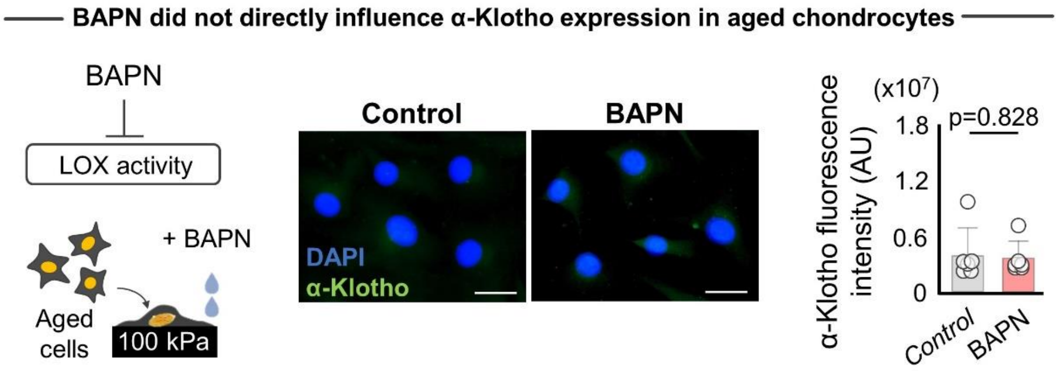

Figure S17. Direct biological impact of BAPN administration on aged chondrocytes

Treatment with BAPN did not influence α-Klotho level in chondrocytes cultured on stiff substrate (n = 6/group). Scale bar: 20μm. Statistical analyses were performed using a two-tailed Student *t*-test. Data are presented as means ± 95% confidence intervals. Source data are provided as a Source Data file.

Table S1. Description of top 10 nuclear morphological features significantly altered by aging

Variable	Description	Illustration
Eccentricity	Ratio of the distance between the foci of the ellipse and its major axis length. Ratio approaching 1 indicates a more elliptical shape.	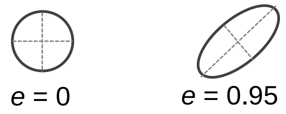
Solidity	Measure of how many holes or concave boundaries in each object. Measured as area/convex hull area. The convex hull area is fitted onto the object by drawing a straight line across straight or concave areas of the cell but expanding outward along object protrusions. Ratio of 1 indicates an object without any concavities or indentations, while a ratio less than 1 indicates an object with holes or an irregular boundary. Ratio of Area to convex hull area.	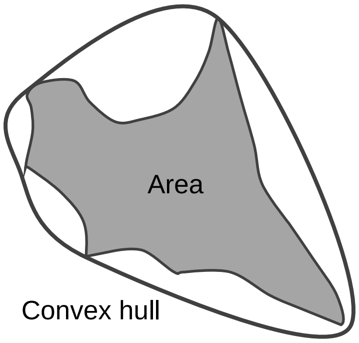
MinorAxisLength	Length of the minor axis of the ellipse that bounds the nucleus/cell.	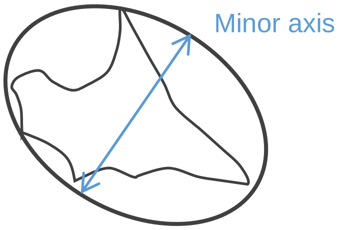
MedianRadius	Median distance of any pixel in the object to the closest pixel outside of the object.	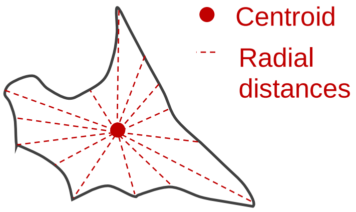
MeanRadius	Mean distance of any pixel in the object to the closest pixel outside of the object.	
MaximumRadius	The greatest distance between any pixel inside the object to the nearest pixel outside of the object.	
Zernike shape features	Set of polynomial coefficients used to describe cell shape with increasing detail. The smallest circle that encloses the object is used to calculate Zernike features. Cells that are more closely related to the Zernike polynomial in question are reflected in a higher value.	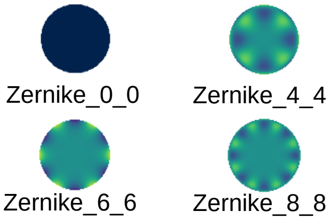

Descriptions were adapted from the CellProfiler documentation[3] and Cutiongco[4].

Table S2. Preparation and characterization of polyacrylamide (pAAm) hydrogels

pAAm	40% Acrylamide (μl)	2% Bis-acrylamide (μl)	50mM HEPES (ul) (pH 8.2)	TEMED (μl)	10% Ammonium persulfate (μl)	Substrate stiffness (kPa)*
5 kPa (soft)	62.5	25	412.5	1.5	5	4.9 ± 0.5
21 kPa (medium)	150	25	325	1.5	5	21.2 ± 0.5
100 kPa (stiff)	150	125	225	1.5	5	100.8 ± 2.1

*The Young's modulus of pAAm gels was obtained by atomic force microscopy.

Table S3. Antibody information and dilutions for immunofluorescence and western blot

Primary antibodies	Host-species	Product number	Dilution
Type II collagen	Rabbit	Ab34712, Abcam	1:200 (cell)
α-Klotho	Rat	MAB1819, R&D systems	1:100 (tissue),<br>1:200 (cell)
α-Klotho	Rabbit	600-401-CF4, Rockland	1:100 (tissue)
Aggrecan	Mouse	MA3-16888, Thermo Fisher	1:200 (cell)
Lamin A/C	Mouse	Sc-376248, Santa Cruz Biotechnology	1:100 (cell)
Lamin B2	Rabbit	Ab151735, Abcam	1:100 (cell)
DNMT1	Rabbit	#5032, Cell Signaling	1:200 (cell)
FAK	Mouse	05-537, Millipore Sigma	1:400 (cell)

Supplemental reference

1. Sebastian A, *et al.* Global Gene Expression Analysis Identifies Age-Related Differences in Knee Joint Transcriptome during the Development of Post-Traumatic Osteoarthritis in Mice. *Int J Mol Sci* **21**, (2020).
2. Chuchana P, *et al.* Secreted α-Klotho maintains cartilage tissue homeostasis by repressing NOS2 and ZIP8-MMP13 catabolic axis. *Aging (Albany NY)* **10**, 1442-1453 (2018).
3. Kamentsky L, *et al.* Improved structure, function and compatibility for CellProfiler: modular high-throughput image analysis software. *Bioinformatics* **27**, 1179-1180 (2011).
4. Cutiongco MF, Jensen BS, Reynolds PM, Gadegaard N. Predicting gene expression using morphological cell responses to nanotopography. *Nature communications* **11**, 1-13 (2020).
